# Transparency in Science Reporting: A Call to Researchers and Publishers

**DOI:** 10.7759/cureus.79493

**Published:** 2025-02-23

**Authors:** Joachim P Sturmberg, Thomas Kühlein

**Affiliations:** 1 College of Health, Medicine and Wellbeing, University of Newcastle, Newcastle, AUS; 2 Research, Central Coast Research Institute, Gosford, AUS; 3 General Practice, Allgemeinmedizinisches Institut, Uniklinikum Erlangen, Erlangen, DEU

**Keywords:** eco-systemic context, evidence-based medicine, nonlinear distribution, patient-centered care, philosophy of medicine, physiological complexity, randomised controlled trials, shared decision-making, statistics, systems and complexity thinking

## Abstract

A recent science communication meeting highlighted a common pitfall in scientific communication: the failure to link the "*what*" - the findings - to the "*so what*" - their real-world implications. The real world is complex, and exploring the complexities of "living world phenomena" requires addressing the interconnectedness and interdependencies of the many variables that shape the patterned outcomes of patient conditions we see in everyday practice. While scientific methods by necessity must simplify complexities, these simplifications should be transparently communicated to foster trust and understanding. Randomised controlled trials (RCTs) aim to eliminate contextual confounders, producing statistically significant average outcomes for a hypothetical "average" patient. While they ensure high internal validity, RCTs often lack external validity, limiting their transferability to real-world practice, where patients differ from the average trial participant. This is an inherent problem of RCTs that cannot be overcome. What is not inherent and should be changed are the outcome elements of the study design and especially their reporting. To achieve "statistical significance", trials use large sample sizes, surrogate and arbitrarily designed composite endpoints, and typically emphasise relative benefits, obscuring absolute benefits, which are often clinically marginal. Transparent reporting of absolute benefits, contextualised to patients’ realities, is crucial for informed, shared decision-making. Patients and clinicians alike must weigh small disease-specific benefits against potential harms, especially when interventions compromise overall well-being or ability to manage daily life circumstances. Transparency matters, it is a moral and ethical imperative. Applied to medical sciences, it is no longer acceptable to argue that the statistical significance of research findings justifies a tacit paternalism that undermines patient autonomy. We propose a transparency framework that could enhance clear and honest communication of research findings - this is crucial to empower both clinicians and patients in making well-informed clinical or public health decisions.

## Editorial

The biological world, including humans and their environment, is inherently complex. Complexity refers to systems where components interact in multiple ways, following local rules that lead to non-linear behaviour, randomness, collective dynamics, hierarchy, and emergence. When tackling complex issues like health, education, the environment, or the economy, humans must inevitably simplify and reduce this complexity to make it more manageable. However, science and its communication must acknowledge the gap between these necessary reductions and the full complexity of the real world.

Science should aim to methodologically address and integrate complexity as much as possible and ensure that the gap between the simplification of knowledge and the complexity of reality is made visible and transparent in its reporting.

**Figure 1:**
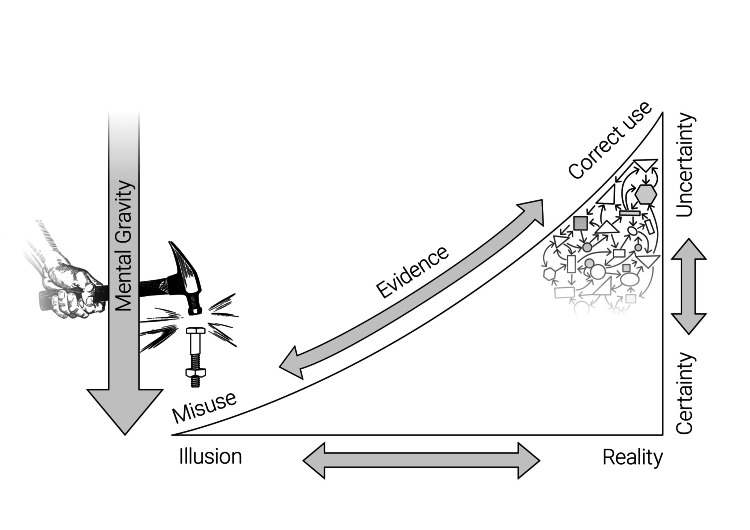
Applying evidence on the continuum between illusion and reality, and certainty and uncertainty. Figure created by the author.

Figure 1 portrays this relationship between the complex reality and the oversimplified version of knowledge or truth we often blindly accept. Complexity, which at least will in its full dimension always exceed human understanding, leaves us in a state of uncertainty that we seek to avoid by simplification to evade anguish and anxiety. The scientific method is regarded as the way to reliably create evidence that helps reduce this uncertainty by generating knowledge to better understand and control the world. In doing so, however, we often oversimplify knowledge to gain an often false sense of certainty, even if it means losing critical information about the world’s actual complexity. This behaviour aligns with the saying, "When all you have is a hammer, everything looks like a nail". "Mental gravity" creates pressure to respond in the easiest possible way. While this tendency may partly be forgivable when driven by human’s need to reduce anxieties, it becomes unacceptable when motivated by vested interests.

The issue lies in the unbridgeable gap between the complexity of reality and the inherent simplification of our knowledge - an issue dating back to at least Galileo’s time. The scientific method [1] has been widely accepted as a trustworthy means of gaining "reliable" knowledge, but we must remain aware that this understanding is limited. By demanding greater transparency in science reporting, we mean that the gap between knowledge and complexity should always be evident, helping us interpret and apply research evidence more carefully. Unfortunately, the ability of science to communicate this limitation, along with its typically incremental findings, is often lacking, contributing to a growing distrust in science [2]. A recent science communication meeting emphasised the danger of failing to connect the "what" with the "so what" [3] - or, in other words, explaining what research findings mean in real-world contexts. This "so what" is crucial for transparency, which, in turn, is essential for building trust in science.

Research design

The nature of a research question dictates its design [4]. A study focused on patient-centred outcomes will require a different approach than one focused on public health or therapeutic effectiveness [5,6]. No single research design is inherently superior; what matters is selecting the right design for the study’s context and goals.

The Greek term phronesis refers to practical wisdom or intelligence, which is central to the task of a clinician. Clinicians must solve practical problems for individual patients, drawing on the best available scientific evidence. This principle lies at the heart of evidence-based medicine (EBM), which its founders defined as "the conscientious, explicit, and judicious use of current best evidence in making decisions about the care of individual patients", or as "integrating individual clinical expertise with the best available external clinical evidence from systematic research" [7]. An old saying goes, "In theory, practice and theory are the same. In practice, they are not", the problem is that practice is more complex than even our best theories. The task is therefore to better align theory and practice. This can only be achieved if theory is reported in a way accessible to clinicians.

Science is a structured endeavour to gain and organise knowledge. Medical sciences focus on optimising patient care, enhancing quality of life and reducing morbidity and premature mortality. For research to be practically useful, research findings must be directly communicated in an accessible way to clinicians. It is not good enough to rely on opinion leaders - often with vested interests - to "translate" research into "the language of clinicians", which can lead to further oversimplification, neglecting important contextual subtleties and nuances.

Randomised controlled trials (RCTs), the prevailing and privileged research methodology for intervention trials, deliberately aim to eliminate contextual variables to avoid bias and confounding. However, RCTs are not always suitable for problems characterised by high clinical variability, such as diabetes [8-10] or glioblastoma multiforme [11,12], or when socioeconomic [13,14] or environmental factors play a significant role like in coronary artery disease [15-19] or cancer [20-23]. While RCTs emphasise internal validity, they often neglect external validity, a problem particularly affecting meta-analyses of RCTs [24]. Knowing the average benefit across a population does not help much in inferring benefits or harms for individual patients with their unique contexts, co-morbidities and personal preferences [24], as no patient precisely matches the average trial participant [25]. As evidenced in some studies, interventions that show average benefits can sometimes cause harm in real-world practice as seen in the Randomized Aldactone Evaluation Study [24,26]. In short, the language of a statistically significant relative average effect of one therapy to another alone obscures what the study results really mean for the individual patient.

Sackett et al. [7], the pioneers of EBM, did not claim that medical decision-making should be based solely on truth, as the truth - particularly regarding individual patient outcomes - will likely remain elusive. Researchers and clinicians alike must accept residual uncertainties, in both research findings and individual patient care. Clinicians must always use the "best available external clinical evidence", but also must understand that while RCTs may be the gold standard for certain research questions, evidence is not as infallible as commonly perceived. This is particularly true for industry-funded trials, which is one reason why they emphasised the need for a critical appraisal of such studies [27-29].

Medical research must acknowledge the complexity of the world and embrace eco-systemic methodologies that are better suited to explore and explain the heterogeneity of diseases and treatment outcomes. Eco-systemic approaches are particularly suited to elucidate how contextual differences influence disease behaviours and thus impact patients’ health and well-being [5,30].

Researchers must recognise that their approaches, by necessity, use simplified models of reality [31]. Transparent reporting must highlight this fact, along with the limitations and implications for individual patient care in clinical practice. Only then will clinicians have the required information best suited to provide individual patients with the practical answers (in the Aristotelian sense of phronesis) they deserve.

Why are study designs and reporting structured the way they are?

This is a critical question requiring thoughtful consideration. A key point is that most medical research, particularly RCTs, is funded by industry rather than independent sources. This creates an inherent conflict, as the overriding goal of the industry is to maximise product sales, equating to maximising profits (an obligation under the Corporations Act), rather than to solely aid personalised decision-making for individual patients. Drug trials published in prominent journals follow a particular pattern: they are large-scale studies involving thousands of participants, recruiting participants from diverse backgrounds, and conducted in multiple centres across multiple countries. The idea behind these kinds of studies is more likely to justify the sale of drugs to as many patients in as many countries as possible, but less to inform clinicians to make the best decisions together with individual patients. While large multi-centre multi-country studies are promoted as leading to the most reliable outcomes, they are plagued by biases arising from variability in context, and the paradox of statistics that larger trials can demonstrate statistical significance for ever smaller but clinically mostly irrelevant outcome differences.

To guarantee statistically significant outcomes for small effect sizes, these trials typically not only need large participant numbers but also need to combine various surrogate and arbitrarily constructed composite endpoints. Both practices can obscure the direct relevance of the findings to individual patients. The choice of multiple centres in many different countries serves the purpose that the results seemingly apply to the whole world but ignore that contextual differences impact how clinicians interpret patient complaints, and how patients respond to treatments. Involving many local researchers who also become the future key opinion leaders to spread the good news primarily serves the purpose to ensure the intervention is applied to as many patients as possible. Moreover, such behaviours also show that these researchers fail to appreciate the differences between statistical and clinical significance. More concerning, the complexities of the real world, arising from the idiosyncrasies of individual patients, are further obscured in meta-analyses, which are regarded as the strongest evidence to justify clinical guideline recommendations. Transparency is further jeopardised when guideline committees include "key opinion leaders" who have received industry funding, raising concerns about potentially biased recommendations.

And lastly, the shift from independent to industry funding has, at least implicitly, fostered practices that align with the interests of funders. These concerns are not criticisms against RCTs and the sound principles of EBM. Instead, they highlight the need to examine how these biases affect clinical practice, which has recently been explored in great detail in the book "The Illusion of Evidence-Based Medicine" [32].

Medical publications overwhelmingly fail the "so what" test

Most medical publications fail to answer the "so what" question clearly. The technical language used in scientific journals is often obscure [1], limiting accessibility for non-scientific readers. Within the medical community, many lack the methodological and statistical knowledge necessary to critically appraise research [33-36], which is mostly a lack of medical education but also of a poorly developed discussion culture in the clinical setting.

This knowledge gap has led to an oversimplification and overreliance on statistical significance, particularly p-values, as a decision-making tool, even though anybody should know that statistical significance is not synonymous with clinical relevance [36-39].

Health professionals clearly require greater research literacy, particularly in understanding the meaning and limitations of statistical significance (a frequentist, not a relational concept [40]) and other statistical information as tools for clinical decision-making. Study results never dictate actions or absolve clinicians from making decisions and being accountable for them. They also do not override a clinician's first duty - *primum non nocere* (first do no harm).

But improvement of communication is possible on both sides. Adopting a transparency framework (Table 1) can guide researchers and publishers - both bear the responsibility of communicating findings in clear, clinically meaningful terms. Research must ultimately support clinicians to answer the question: "What does this mean for the patient in front of me?" [25].

**Table 1:** Transparency framework. NNT: number needed to treat; NTN: number treated needlessly; ITI: index of therapeutic impotence; *: the percentage of patients treated without receiving a benefit (ITI = NNT-1/NNT) [41].

Domain	Are the important issues clearly described?		Comments		
Context	Clinical setting(s), e.g., hospital, individual practice, and community		Each context will have its own unique characteristics that influence the dynamics of patient and service behaviours		
	Geography, e.g., urban, rural, regions within a country, and multiple countries		Geography is associated with the distribution of social determinants of health		
			Multi-country settings require the reporting of individual outcome results in addition to global outcomes		
Patients	Should be clustered according to similarities in characteristics. This will allow the identification of the impact of those features associated with differential outcomes within a study group		Aggregate demographic characteristics can mask the impact of differences in demographic features		
Assumptions	What is the a priori expected minimally clinically important difference (MCID)		Even large percentage change differences may be clinically unimportant		
Reporting	Clinicians require information about the absolute benefits and harms identified		Typical reporting only provides relative differences which are often statistically significant, but not clinically meaningful, e.g., relative benefit/harm, hazard ratios, Kaplan-Meier curves, events per 1000 patient-years, etc.		
					The heterogeneity in outcome differences is highlighted and explained		While an outcome may be clinically unimportant for the total population, it may still have benefits for a small group of patients		
	Subgroups experiencing greater benefit/harm are highlighted and reasons are provided							
	Transparent reporting of relative and absolute benefits of an intervention taking into account the prevalence of a condition. While the relative benefit remains unchanged, the absolute benefit/NNT changes dramatically				
	Prevalence	3:1,000	30:1,000	300:1,000	
	Benefit of intervention	1:1,000	10:1,000	100:1,000	
	Relative benefit	33%	33%	33%	
	Absolute benefit	0.01%	0.10%	1%	
	NNT	1,000	100	10	
	NTN	999	99	9	
	ITI^*^	99.99%	99.90%	99%	
	Relevance	What does the outcome add to our current knowledge/understanding?		Understanding these issues is fundamental to informed shared decision-making between clinicians and patients		
How will/should the outcomes influence clinical care?					
How do the outcomes help in clinical decision-making?					
The heterogeneity in outcome differences is highlighted and explained					
How do the outcomes impact patients’ quality of life?					
How does the implementation of the outcomes affect the patient’s treatment burden?					

What does clear communication entail?

The manner in which research results are presented has a marked influence on clinicians’ judgement of intervention benefits [42]. Hence, transparent communication must go beyond the clear methodological description [43-45], it requires abandoning the misleading and/or confusing use of relative benefits and harms, hazard ratios, Kaplan-Meier curves, events per 1000 patient years, and others. Instead, benefits and harms should be conveyed in absolute terms, e.g., how many out of 100 patients benefited/were harmed, with full consideration of the context in which the findings were observed. Outcomes must emphasise clinically relevant endpoints [46] rather than composite endpoints that may obscure the true meaning for patients [47].

To be transparent, survival data must be reported in terms of all-cause mortality - emphasising solely a reduction in disease-specific mortality while no reduction in total mortality is achieved is clearly neither transparent nor ethical.

At a time where we value patient-centred care and shared decision-making, we should know whether or not an intervention has achieved a minimally clinically important difference (MCID), i.e., was the outcome meaningful from a patient perspective [48], and what impact it had on their quality of life [49]. Successful disease-specific outcomes may well result in a significant decline in overall quality of life [50]. While both measures are subjective, these data are important to provide the "best possible" guidance for "the patient in front of us" [25].

Transparency is not just a scientific obligation, it is a moral and ethical one [44,51]. It is no longer acceptable to justify interventions based solely on the statistical significance of relative outcome differences. Tables 2-7 provide examples that illustrate the differences between the reported findings of published trials and how these findings could have been presented in a transparent way (note: they have not been reviewed for methodological integrity (e.g., [52]) or conflicts of interest (e.g., [53])). The last row in each table lists potential points that transparent reporting might have emphasised. These comments have been included without considering broader ethical implications; however, these would need to be highlighted, particularly when such conclusions reinforce a paternalistic approach to healthcare [51].

**Table 2:** Examples from the literature - drug trial. Note: All tables have been compiled using the outcomes data (columns "Outcomes per 100 people") provided in the original articles. All other data have been calculated from these data by the authors. Missing outcomes, where possible, were calculated. Studies have not been reviewed for methodological integrity or conflicts of interest. To distinguish "Benefits" from "Harms/Adverse Outcomes", the latter are presented in *italic* font. (1) Benefits reported as RRR – relative risk reduction (at the end of a study period). (2) *Harms* reported as RH – relative harm. (3) Sig - statistical significance. (4) NNT – number needed to treat (rounded); *NNH* – number needed to harm. (5) NTN – number treated needlessly (NTN = NNT-1), - figures = benefit; *NTWH* – number treated without harm (NTWH = NNH-1), - figures = less harm. (6) ITI – index of therapeutic impotence (ITI = NTN/NNT*100), ITI > 100 = benefit; *ITH* – index of therapeutic harm (ITH = NNH/NTWH*100 - 100), ITI > 100 = less harm. $ One patient developed contact dermatitis; $$ significant differences between clusters. BMI: body mass index; nr: not reported; ns: not significant.

Study focus	Drug trial [54]								
Title (Year)	Once-weekly semaglutide in adolescents with obesity (2022)								
Context	Adolescents (12 to <18 years of age) with (a) obesity (a body-mass index (BMI) in the 95th percentile or higher) or (b) overweight (a BMI in the 85th percentile or higher) and at least one weight-related coexisting condition.								
Results	Outcomes measured	Outcomes per 100 people		Benefits^(1)^/*Harms*^(2)^ RELATIVE (%)	Sig^(3)^	Transparent reporting			
						Benefits/*Harms *ABSOLUTE (%)	Benefit/*Harm *Comparison		
		Study group	Control group				NNT^(4)^ *NNH*	NTN^(5)^* ITH*	ITI^(6)^ *ITH*
	Change in BMI (%)	-16.1	0.6	-	YES	-	-	-	-
	≥ 5%	72.5	17.7	-308.7	nr	-55.8	1.8	2.8	154.8
	≥ 10%	61.8	8.1	-666.7	nr	-53.7	1.9	1.9	153.8
	≥ 15%	53.4	4.8	-1004.3	nr	-48.6	2.1	3.1	148.6
	≥ 20%	37.4	3.2	-1059.5	nr	-34.2	2.9	3.9	134.2
	Any adverse event	89.2	88.7	9.6	nr	-8.6	11.7	12.7	108.6
	Serious adverse event	11.5	9.7	18.3	nr	-1.8	56.4	57.4	101.8
Conclusions	Among adolescents with obesity, once-weekly treatment with a 2.4-mg dose of semaglutide plus lifestyle intervention resulted in a greater reduction in BMI than lifestyle intervention alone.								
What would transparent, i.e., clinically relevant, reporting have emphasised?									
· Significantly more participants achieved ≥ 5% change in BMI.									
Note: The study group was 7.3 kg heavier, BMI difference: 2 (37.7 vs. 35.7).									
· Greater weight loss was achieved while on the medication.									
· Unclear if there was a difference between obese and overweight kids.									
· Rapid weight gain within 7 weeks of end-of-trial in the treatment group.									

**Table 3:** Examples from the literature - medication dosage effects. To distinguish "Benefits" from "Harms/Adverse Outcomes", the latter are presented in italic font. (1) Benefits reported as RRR – relative risk reduction (at the end of a study period). (2) Harms reported as RH – relative harm. (3) Sig - statistical significance. (4) NNT – number needed to treat (rounded); NNH – number needed to harm. (5) NTN – number treated needlessly (NTN = NNT-1), - figures = benefit; NTWH – number treated without harm (NTWH = NNH-1), - figures = less harm. (6) ITI – index of therapeutic impotence (ITI = NTN/NNT*100), ITI > 100 = benefit; ITH – index of therapeutic harm (ITH = NNH/NTWH*100 - 100), ITI > 100 = less harm. LDL: low-density lipoprotein; MI: myocardial infarction; CHD: coronary heart disease.

Study focus	Medication dosage effects [55]								
Title (Year)	Intensive lowering of LDL cholesterol with 80 mg versus 20 mg simvastatin daily in 12,064 survivors of myocardial infarction: a double-blind randomised trial (2010)								
Context	Men and women, aged 18-80 years, who had a myocardial infarct.								
Results	Outcomes measured	Outcomes per 100 people		Benefits^(1)^/*Harms*^(2)^ RELATIVE (%)	Sig^(3)^	Transparent reporting			
						Benefits/*Harms *ABSOLUTE (%)	Benefits/*Harm *Comparison		
		Study group	Control group				NNT^(4)^ *NNH*	NTN^(5)^ *ITH*	ITI^(6)^ *ITH*
	Non-fatal MI	6.6	7.7	14.2	Yes	1.09	92	91	98.91
	Revascularisation	9.5	10.1	6.5	ns	0.66	152	151	99.34
	Stroke	4.2	4.5	8.6	ns	0.4	251	250	99.6
	CHD-death	7.4	7.3	-1.9	ns	-0.1	740	741	0.13
	All-cause mortality	16	16.1	0.6	ns	0.1	1062	1061	99.91
	Myopathy	1.4	0.2	583	Yes	1.2	86	85	98.8
Conclusions	The 6% (SE: 3.5%) reduction in major vascular events with a further 0.35 mmol/L reduction in LDL cholesterol in our trial is consistent with previous trials. Myopathy was increased with 80 mg simvastatin daily, but intensive lowering of LDL cholesterol can be achieved safely with other regimens.								
What would transparent, i.e., clinically relevant, reporting have emphasised?									
· Higher dose statin use has no impact on all-cause mortality over usual dose statins.									
· Higher dose statin use is associated with a statistical and clinically relevant increase over usual dose statins in myopathy.									

**Table 4:** Examples from the literature - disease-specific invasive intervention - coronary artery disease. To distinguish "Benefits" from "Harms/Adverse Outcomes", the latter are presented in italic font. (1) Benefits reported as RRR – relative risk reduction (at the end of a study period). (2) Harms reported as RH – relative harm. (3) Sig - statistical significance. (4) NNT – number needed to treat (rounded); NNH – number needed to harm. (5) NTN – number treated needlessly (NTN = NNT-1), - figures = benefit; NTWH – number treated without harm (NTWH = NNH-1), - figures = less harm. (6) ITI – index of therapeutic impotence (ITI = NTN/NNT*100), ITI > 100 = benefit; ITH – index of therapeutic harm (ITH = NNH/NTWH*100 - 100), ITI > 100 = less harm. LDL: low-density lipoprotein; CHD: coronary heart disease.

Study focus	Disease-specific invasive intervention [56]								
Title (Year)	Survival after invasive or conservative management of stable coronary disease (2023)								
Context	5179 original ISCHEMIA trial participants were included with a median age of 65 years, 23% women, 16% Hispanic patients, 4% Black patients, 42% with diabetes, and a median ejection fraction of 0.60.								
Results	Outcomes measured	Outcomes per 100 people		Benefits^(1)^/*Harms*^(2)^ RELATIVE (%)	Sig^(3)^	Transparent reporting			
						Benefits/*Harms *ABSOLUTE (%)	Benefit/Harm Comparison		
		Study group	Control group				NNT^(4)^ *NNH*	NTN^(5)^ *ITH*	ITI^(6)^ *ITH*
	CHD-death	5.7	7.6	24.9	YES	1.9	53	52	98.11
	All-cause mortality	10.6	10.9	3.1	ns	0.33	297	296	99.66
		-	-	OR: 0.98	-	-	-	-	-
	Major event	6	-	9.1	ns	0.6	172	171	99.42
	All-cause mortality	nr	-	nr	-	nr	-	-	-
Conclusions	Studies of patient preferences demonstrate that quality of life, functional status, and survival rank highly. We have previously shown that quality of life was improved with an initial invasive strategy, and the extent of benefit was related to the degree of angina on a medically tolerated regimen. Those without angina did not experience quality-of-life benefits. We believe the data from this interim follow-up report demonstrating no difference in survival between groups at seven years will add to the evidence base for shared decision-making between patients and their physicians.								
What would transparent, i.e., clinically relevant, reporting have emphasised?									
· While invasive management statistically reduces CHD-death, all-cause mortality is not reduced.									
· The statistically significant reduction in CHD risk is at best of slight clinical importance.									

**Table 5:** Examples from the literature - prevention trial - aspirin in the elderly. To distinguish "Benefits" from "Harms/Adverse Outcomes", the latter are presented in italic font. (1) Benefits reported as RRR – relative risk reduction (at the end of a study period). (2) Harms reported as RH – relative harm. (3) Sig - statistical significance. (4) NNT – number needed to treat (rounded); NNH – number needed to harm. (5) NTN – number treated needlessly (NTN = NNT-1), - figures = benefit; NTWH – number treated without harm (NTWH = NNH-1), - figures = less harm. (6) ITI – index of therapeutic impotence (ITI = NTN/NNT*100), ITI > 100 = benefit; ITH – index of therapeutic harm (ITH = NNH/NTWH*100 - 100), ITI > 100 = less harm.

Study focus	Prevention trial [57]								
Title (Year)	Low-dose aspirin and the risk of stroke and intracerebral bleeding in healthy older people: secondary analysis of a randomized clinical trial (2023)								
Context	Secondary analysis of the Aspirin in Reducing Events in the Elderly (ASPREE) randomized, double-blind, placebo-controlled trial of daily low-dose aspirin among community-dwelling people living in Australia or the US older adults free of symptomatic cardiovascular disease. Recruitment between 2010 and 2014, follow-up for a median (IQR) of 4.7 (3.6-5.7) years; analysis completed between August 2021 and March 2023.								
Results	Outcomes measured	Outcomes per 100 people		Benefits^(1)^/*Harms*^(2)^ RELATIVE (%)	Sig^(3)^	Transparent reporting			
						Benefits/*Harms *ABSOLUTE (%)	Benefit/*Harm *Comparison		
		Study group	Control group				NNT^(4)^ *NNH*	NTN^(5)^ *ITH*	ITI^(6)^ *ITH*
	All stroke	2.05	2.11	3.3	ns	0.07	1433	1432	99.93
	Ischaemic stroke	1.53	1.73	11.5	ns	0.19	504	503	99.8
	Haemorrhagic stroke	0.51	0.39	33.3	ns	0.13	777	776	99.87
	All intracranial bleeds	1.13	0.82	37.6	Yes	0.31	322	321	99.69
	Fatal bleeds	0.3	0.24	26.9	Yes	0.06	1548	1547	99.93
	All-cause mortality	nr	-	-	-	-	-	-	-
	Subgroup variability	-	-	-	-	-	-	-	-
	· Age 65-74	-	-	HR 2.11	-	-	-	-	-
	· Age 75-84	-	-	HR 1.19	-	-	-	-	-
	· Age ≥ 85	-	-	HR 1.03	-	-	-	-	-
	· Frail	-	-	HR 0	-	-	-	-	-
	· Prefrail	-	-	HR 0.96	-	-	-	-	-
	· Not frail	-	-	HR 1.74	-	-	-	-	-
Conclusions	This study found a significant increase in intracranial bleeding with daily low-dose aspirin but no significant reduction in ischemic stroke. These findings may have particular relevance to older individuals prone to developing intracranial bleeding after head trauma.								
What would transparent, i.e., clinically relevant, reporting have emphasised?									
· The findings are presented as incidence over time (No./1,000 patient-years), which has limited utility for shared decision-making with individual patients.									
· Overall, aspirin has no clinically relevant benefit.									
· Clinically important subgroup analysis shows that the greatest risk of aspirin use is for younger and non-frail patients.									

**Table 6:** Examples from the literature - population health. To distinguish "Benefits" from "Harms/Adverse Outcomes", the latter are presented in italic font. (1) Benefits reported as RRR – relative risk reduction (at the end of a study period). (2) Harms reported as RH – relative harm. (3) Sig - statistical significance. (4) NNT – number needed to treat (rounded); NNH – number needed to harm. (5) NTN – number treated needlessly (NTN = NNT-1), - figures = benefit; NTWH – number treated without harm (NTWH = NNH-1), - figures = less harm. (6) ITI – index of therapeutic impotence (ITI = NTN/NNT*100), ITI > 100 = benefit; ITH – index of therapeutic harm (ITH = NNH/NTWH*100 - 100), ITI > 100 = less harm.

Study focus	Population health [58]								
Title (Year)	Vitamin D supplementation and major cardiovascular events: D-Health randomised controlled trial (2023)								
Context	Monthly supplementation of older adults (60-84 years) with a monthly dose of 60,000 IU of vitamin D after a major cardiovascular event in Australia between 2014 and 2020.								
Results	Outcomes measured	Outcomes per 100 people		Benefits^(1)^/*Harms*^(2)^ RELATIVE (%)	Sig^(3)^	Transparent Reporting			
						Benefits/*Harms *ABSOLUTE (%)	Benefit/*Harm *Comparison		
		Study group	Control group				NNT^(4)^ *NNH*	NTN^(5)^ *ITH*	ITI^(6)^ *ITH*
	Major event	6	6.6	9.1	ns	0.6	172	171	99.42
	All-cause mortality	nr	-	nr	-	nr	-	-	-
Conclusions	Vitamin D supplementation might reduce the incidence of major cardiovascular events, although the absolute risk difference was small and the confidence interval was consistent with a null finding. These findings could prompt further evaluation of the role of vitamin D supplementation, particularly in people taking drugs for the prevention or treatment of cardiovascular disease.								
What would transparent, i.e., clinically relevant, reporting have emphasised?									
· Unlikely to be of clinical relevance as a population health measure.									

**Table 7:** Examples from the literature - treatment response variability. To distinguish "Benefits" from "Harms/Adverse Outcomes", the latter are presented in italic font. (1) Benefits reported as RRR – relative risk reduction (at the end of a study period). (2) Harms reported as RH – relative harm. (3) Sig - statistical significance. (4) NNT – number needed to treat (rounded); NNH – number needed to harm. (5) NTN – number treated needlessly (NTN = NNT-1), - figures = benefit; NTWH – number treated without harm (NTWH = NNH-1), - figures = less harm. (6) ITI – index of therapeutic impotence (ITI = NTN/NNT*100), ITI > 100 = benefit; ITH – index of therapeutic harm (ITH = NNH/NTWH*100 - 100), ITI > 100 = less harm. * One patient developed contact dermatitis; ** significant differences between clusters.

Study focus	Treatment response variability [59]								
Title (Year)	Differential response to scrambler therapy by neuropathic pain phenotypes (2021)								
Context	Pain clinic setting - patients with chronic neuropathic pain of various aetiologies. Post-hoc cluster analysis of the Neuropathic Pain Symptom Inventory (NPSI) profiles to identify subgroups of patients regarding neuropathic pain phenotypes and treatment outcomes. The aetiology of chronic pain has been detailed; the nature of pain (superficial, deep, paroxysmal, evoked, and paresthesia) and pain severity have been described with three distinct pain pattern clusters identified, and outcomes were reported for each cluster.								
Results	Outcomes measured	Outcomes per 100 people		Benefits^(1)^/*Harms*^(2)^ RELATIVE (%)	Sig^(3)^	Transparent reporting			
						Benefits/*Harms *ABSOLUTE (%)	Benefit/*Harm *Comparison		
		Study group	Control group				NNT^(4)^ *NNH*	NTN^(5)^ *ITH*	ITI^(6)^ *ITH*
	Overall pain reduction	-15%	-	3.7^*^	ns	-	Cannot be calculated from the data presented		
	*· *Cluster 1	-18%	-	-	YES^**^	-	-		
	*· *Cluster 2	-23%	-	-	-	-	-		
	*· *Cluster 3	-3.70%	-	-	-	-	-		
Conclusions	Treatment response to scrambler therapy appears different depending on the neuropathic pain phenotypes, with more favourable outcomes in patients with preferentially paroxysmal pain rather than persistent pain.								
What would transparent, i.e., clinically relevant, reporting have emphasised?									
· Heterogeneity of neuropathic pain has been identified.									
· Neuropathic phenotype is associated with treatment response.									
· Important to identify pre-treatment neuropathic phenotype.									

Conclusions

The complexity of biological systems and human health necessitates a more nuanced approach to research design, data interpretation, and communication of findings. Eco-systemic approaches better capture the contextual differences in disease behaviour and treatment responses compared to the reductionist trial designs.

Reporting absolute benefits and harms, clinically relevant endpoints, and patient-centred outcomes is a *sine qua non* to transparency and provides the foundation for honest discussions about the clinical relevance of findings, thereby supporting the goal of well-informed, shared decision-making in clinical practice. The proposed transparency framework could function as both an educational tool to enhance clinicians’ ability to interpret and critically assess trials and guideline recommendations as well as a guide to address the "so what" question. By doing so, it helps make research findings accessible and meaningful to clinicians and patients, empowering them to make the best possible shared clinical decisions while fully considering their unique personal context.
